# Auscultation and Thoracic Diseases

**Published:** 1826-04

**Authors:** 


### XIII.

### AUSCULTATION AND THORACIC DISEASES.

1. *An Introduction to the Use of the Stethoscope; with its Application to the Diagnosis in Diseases of the Thoracic Viscera; including the Pathology of these various Affections.* By WILLIAM STOKES, M.D. 8vo. pp. xiv. 226. Edinburgh, London, and Dublin, 1825.
2. *Dissertatio Medica Inauguralis, quædam de Auscultatione Mediata complectens.* Auctore GULIELMO BENNET, A.B. Trin. Coll. Dub. 8vo. Edinburgi, 1825.
3. *A Treatise on the different Methods of Investigating the Diseases of the Chest, particularly Percussion, and the Use of the Stethoscope. Translated from the French of M. Collin, &c.* By W. N. RYLAND, M.D. 8vo, pp. 67, with Plates. Burgess & Hill, 1825.

WE were the first in this country to introduce the subject of auscultation, (January, 1820) through the medium of an extensive review of Laënnec's work, and we are happy to see that the subject is at last studied by the rising generation of the profession, with that zeal which will ultimately, and at no distant period, lead to a general adoption of those principles of exploration, so successfully employed on the Continent, for the investigation of thoracic diseases. It was Baglivi, we believe, who deplored so pathetically the difficulty of ascertaining the nature of diseases of the lungs. Yet there is no other internal organ whose disorders are now so easily determined as the organ of respiration. Still there is but a small proportion of practitioners in general, who give themselves the trouble of studying auscultation and percussion—and fewer still of those who are above 45 years of age. On the latter class we cannot expect to make much impression. Their habits are confirmed—their routine established—their opinions almost irrevocably fixed—and last, not least, they are unwilling to go a second time to school. It appears to us, however, that the field of investigation which has been opened by Laënnec, is so capable of beneficial cultivation, that prejudice itself, as well as apathy, must give way, in no long time, and the practice of auscultation become general. Were we not fully and conscientiously convinced of the truth of this prediction, we should not take the pains we do, to impress on the minds of our brethren the necessity of bestirring themselves, in order that the stigma of indolence (to give it the mildest term) may be removed from the practitioners of these Islands. On this account we shall brave the taunts of the prejudiced and the ignorant, on the present occasion, and enter more fully than we yet have done, on the interesting subject under review. We are satisfied that the castle of indolence requires repeated assaults before its towers shall fall to the ground; and we will not cease our fire till the good work is accomplished.

Gutta cavat lapidem non vi sed sæpe cadendo.

With this brief preface we shall proceed to an account of the first work at the head of this article.

With that modesty which is ever an attribute of the ingenuous and philosophic mind, Dr. S. professes his highest aim, in this "introduction," to be that of an assistant to the student, whether he be a pupil commencing his medical labours or one advanced to practice, and wishing to become acquainted with the new methods of diagnosis in diseases of the chest. He adverts to the objection; that, as we are already in possession of the excellent works of Professor Laënnec and Dr. Forbes on the same important subject, the present Essay will be held to have been unnecessary; it appears to him, however, that in neither of these works have the accounts of the different signs obtained by the stethoscope been properly connected with that of the pathological state of the viscera in which they originate. He has, therefore, after describing each phenomenon, endeavoured to give a succinct view of the morbid condition producing it; so that, upon perceiving any deviation from the natural state, we may, on comparing it with the description of the phenomenon which it most resembles, be at once led to that of the alteration which is to be expected. The phenomena obtained by examining the respiration, voice, and action of the heart are distributed into two heads—the natural and pathological. Under the pathological manifestations of respiration, an account is given of the different *râles*,* and following each, the pathological state of the part from which it arises: thus, under the *crepitous wheeze*, is given the pathology of pneumonia, œdema of the lung, and pulmonary apoplexy: under the *sonorous wheeze*, that of bronchitis; and, under the hissing wheeze, that of emphysema: when we come to the pathological phenomena of the voice, we have phthisis under *pectoriloquy*; pleurisy under *egophony*; and pneumothorax under the *metallic tinkling*. Dr. S. has preferred this minute pathology, under the conviction that, as morbid appearances are so different from one another, their division in this way, especially when connected with the symptoms, is proper and desirable. After describing each disease, he has inserted a case illustrative of the use of the stethoscope in that particular affection; and, in the Appendix, an account of pulmonary gangrene and a condensed translation of Dr. Andral's admirable Thesis on Expectoration, are subjoined: his pathology is taken chiefly from the works of this physician and Laënnec, "to which

* RALE. Ere long, this ill-sounding word will find its way to the drawing-room; the very atmosphere of which seems to be endowed with the quality of rendering ugly things pretty; and, in due time, we shall have to listen, with approving complaisance, to the philosophy of the *rails*, as it flows instinctively from the lips of languishing ladies. *Râle*, as a primitive French monosyllable, has an exact analogy with our own expressive Anglo-Saxon word *wheeze*, which signifies "to breathe with noise." With us, who are plain persons, *wheeze*, *s.* shall in this article denote the sound simply that is produced by the acts of respiration; and its modifications shall, at the same time, be expressed by the addition of qualifying terms· besides, we shall employ *wheezing*, *s.* to represent the act of breathing or performing respiration with an audible sound; as it always is, however, faint that sound may be.

last great name," he says, with the elegance of a generous nature, "it is almost presumption to offer any thing like praise."

Without going into a dissertation on the utility of the stethoscope, Dr. S. states in explicit terms that, besides its vast importance in the diagnosis of a most difficult class of diseases, the instrument *does* lead to many useful *practical* results. In pneumonia and pleurisy, he says, a daily examination with the stethoscope, points out the progress of the disease, its exact seat, the effect of remedies, and the necessity of their repetition, or the advantage of their omission. In circumscribed pleurisy, in wounds of the thorax, he adds, perhaps rather strongly, its utility is undeniable. From ignorance of its application, he proceeds to say, misplacement of the heart arising from pleuritic effusion, has been mistaken for dilatation of that organ, while the original disease was entirely overlooked. By misapprehension also, pleurisy has been viewed as rheumatism, and a critical diaphoresis checked in pneumonia: in confirmed phthisis, when the hopes of the sufferer's friends are excited by an ignorant practitioner, the physician, with the aid of a stethoscope, has at least the melancholy advantage of saving to those friends the pangs of disappointed hope, and to the patient himself the torture of unavailing remedies. By means of this instrument, latent inflammatory affections of the lungs can be detected long before they have become evident from their external symptoms. These are the cases, indeed, where a practitioner, ignorant of *mediate auscultation*, would be exceedingly embarrassed. Our zealous advocate for stethoscopy believes he could adduce a host of other instances, but refrains from doing so, in the firm conviction, that such will not be required by any one who has adopted the practice in a few cases of thoracic disease. Even without reference to actual disease, he asks, is it not a great practical advantage, that in doubtful cases we can explore the hidden recesses of the chest, and say with confidence, "*there is no disease here?*"—From these general observations, we should pass, with Dr. Stokes, to his preliminary remarks on the advantages of percussion and auscultation, as means of ascertaining the effects of morbid action on the parts contained within the chest; but, from the full explanation of these processes given in the Number of this Journal for December, 1823, p. 569, and in that for January, 1825, pp. 66, 79, we deem an additional reference to the subject as being at present unnecessary; we may observe, however, that Dr. Stokes' description of them is elegant, concise, and accurate.

At p. 13 of his volume, Dr. S. proceeds to explain the phenomena of respiration, and these he divides, according to his plan, into the natural and pathological. As, on former occasions, we have not entered very minutely into a consideration of this important topic, we now propose retracing the Doctor's view of it, which, in our judgment, is exceedingly just and comprehensive.

Natural Phenomena of Respiration. These may be divided into two classes, *viz.* those that relate to the motions of the chest, and those which arise from particular modifications of the respiratory murmur.

In a healthy person, uninfluenced by any action or passion, inspiration and expiration should be performed slowly and regularly, without the painful effort of any muscle; their rhythm should be constant and uniform; all the ribs should be elevated; and the dilatation and contraction, unless in cases of deformity, should be equally marked on both sides. In different individuals, the succession of the respiratory motions varies: in general, there are twenty respirations in the minute, and every fifth is the strongest. In women, children, and weak individuals, its frequency is greater; the passions, exercise, and repose, the quality of the air, and a multitude of other circumstances, cause the rhythm to undergo constant variations: during sleep respiration is less frequent and deeper ; when performed by the intercostal and other respiratory muscles, it is called *thoracic;* when by the action of the diaphragm alone, *abdominal.* Its sound varies according to the different parts of the chest examined, the frequency of breathing, and the particular conformation, age, or sex of the individual. When we apply the stethoscope to the chest of a healthy person, we hear, during respiration, a slight but very distinct murmur : this indicates the entrance of the air into the cells of the lung, and its expulsion ; it is nearly equally strong at every point of the chest, but most so where the lungs are nearest the surface, *i. e.* in the superior-lateral and postero-inferior parts : it is best heard at the arm-pit and the space comprised between the clavicle and edge of the trapezius muscle ; over the larynx, trachea, and rest of the lungs, it is distinct ; but, at the same time, has a particular character which enables us to perceive that the air is passing through a canal of greater diameter than the cells of the lungs. In these situations, the expansion of the pulmonary tissue is not distinguishable, and the air seems, during inspiration, to be drawn in through the cylinder ; during expiration, to issue from it : the sound of this respiration, which is called *tracheal,* may be compared to that produced by a pair of bellows.-Inspiration, when slow or deep, is sometimes scarcely heard : hence it often becomes necessary to desire the person to breathe quickly and strongly ; the murmur of respiration is distinct and sonorous, in children, women, and men of an irritable habit ; the expansion of the air-cells is more perceptible ; and the sensation is such, that these appear to be more dilated than in the lungs of a healthy man. This difference of sound is best perceived during inspiration, and is more marked as the person is younger : it generally remains until puberty, or a little beyond that age. In adults, the respiratory murmur varies much : in many who are healthy, it is scarcely heard, unless when they make a strong inspiration ; and, in these, the breathing is generally frequent : in others, on the contrary, it is distinct, and similar to that of infants : such individuals seem more disposed to diseases of the pulmonary organs.

PATHOLOGICAL PHENOMENA OF RESPIRATION. Like the *natural,* these may be considered under two heads,—those which relate to the motions of the chest, and those derived from the character of the respiratory murmur.

In disease, the thoracic motions are either frequent or unfrequent, quick or slow, regular or irregular, great or small, equal or unequal, free or difficult, complete or incomplete, abdominal or thoracic. In order, says Dr. S. to observe and appreciate these differences, it is proper that the patient, if his strength permits it, be made to sit up with both the arms hanging down by the sides of his chest, which should be uncovered, so that nothing shall interfere with the muscular power producing respiration : but, he adds, the most usual alterations are sufficiently evident to render these precautions, in many instances, unnecessary.

Respiration is *frequent* when, in an adult, it is performed more than 18 or 20 times in a minute : it is *unfrequent,* when fewer take place. This *frequency* is natural to infants, females, and persons of a nervous temperament : it occurs after exercise and strong emotions ; in hot climates, and in very elevated situations, from the smaller quantity of oxygen being inspired in a given volume of air : and it is observed, independently of any thoracic affection, in worm-disorders, in *all* spasmodic complaints, *(does experience confirm this ?)* and generally in the whole class of inflammatory diseases. *Unfrequent* respiration is usually seen only in the comatose and hysteric seizures, or during the last moments of life ; it proceeds from suspension of the nervous influence and weakening *(disbalancing rather)* of the muscular powers; the *frequent*, is caused by pain in the chest *(pain of any kind)*, every obstacle to the free circulation of air in the bronchial tubes, and every alteration which renders the pulmonary tissues unfit for respiration.

Respiration is *quick*, when the acts of breathing are short, rapid, and sudden; *slow*, when they are long and gradual; *accelerated*, when both quick and frequent; and it is *panting*, when much accelerated and irregular. Quick respiration, says our author, is sometimes met in alliance with the unfrequent variety among some robust subjects, also in acute diseases, and in the last moments of life. The quickness of breathing appears to arise from the same causes as its frequency; its slowness is observed under the same circumstances as its unfrequency, which it often accompanies.

Respiration is *regular*, when the inspirations and expirations succeed at equal intervals; it is *irregular*, when these intervals are differently prolonged; *intermittent*, when one or more inspirations take place later, or fail altogether; and *interrupted*, when the expiration seems to take place before the inspiration was finished. These different states are met with in the thoracic and abdominal inflammations, and also in the nervous affections.

Respiration is *great*, when a perfect expiration is succeeded by a slow or quick inspiration, accompanied by a great enlargement of the chest: it is *small*, when the dilatation is scarcely perceptible. Great and unfrequent respiration is observed especially in cerebral fevers at the approach of phrenitic delirium; its smallness is most frequently a symptom of affections of the chest.

Respiration is *high*, when the chest remains elevated, the inspiration not having been preceded by a complete expiration: this occurs in pneumonia, when the breathing is frequent, small, and quick.

Respiration is *equal*, when the inspiration, whether great or small, quick or slow, is followed by a similar expiration; *unequal*, when either of these motions is stronger than the other, or more prolonged: the typhoid fevers, and most of the spasmodic affections and asthmas, present examples of it; and it is a constant symptom in acute pneumonia and pleurisy. When the pleura is inflamed, inspiration is quick; and the expiration, although very short, appears long when compared with the inspiration; the seat of the pain in this disease accounts easily for the phenomenon: when, on the contrary, the lung is inflamed, expiration is the shorter of the two; this motion, which cannot be performed without painfully compressing the affected organ, seems hardly to take place, so that the chest remains always elevated; high respiration, then, depends on the difficulty of expiration.

Respiration is *free*, when it is performed without laborious effort: it is difficult, when, in its production, the great accessory muscles are called into action, or when the proper inspiratory muscles contract with force, and, as it were, convulsively. This difficulty of respiration may sometimes be perceived by mere inspection of the neck; we then see the scaleni muscles hard, prominent, and trembling; and the same appearance is observed in the intercostal muscles of an emaciated patient. Of this state of respiration, there are different degrees from the difficult to the suffocating: in this last, the patient, threatened with immediate suffocation, cannot remain in the horizontal position; sitting and bending himself forward, he seeks a solid support for his hands, and thus, fixing the superior extremities, he painfully contracts the great muscles of respiration, the whole effort of which is concentrated on the chest, which is greatly dilated. This state is called orthopnœa; and is frequently present in the attack of asthma, in diseases of the heart, and, sometimes, becomes habitual in persons affected with emphysema of the lung. Most of the thoracic maladies, indeed, and a great number of the abdominal, give rise to this state: thus, every obstacle to the entrance of air into the lungs, or to the dilatation of the chest, whether it does or does not exist in this cavity, may equally be the cause of difficult respiration.

Respiration is *complete* when produced by the equal concurrence of the two lungs: it is characterised by the extent of motion of the chest, and its equality of force: it is *incomplete*, when one side of the thorax remains immoveable, either wholly or in part, or at least partakes of much less motion than the opposite. This is one of the most constant and certain symptoms furnished by examination of the thoracic movements: it belongs, almost exclusively, to diseases of the chest, and is often sufficient of itself to point out the existence of pleurisy or pneumonia in infants; and, in all cases, by indicating at once the seat of the disease, saves the patient much useless questioning and fatiguing examination: it depends, occasionally, on an inflammation of the lung, sometimes on pleurisy, sometimes on simple pleurodyné. Individuals often exhibit this phenomenon, although enjoying perfect health; but it is then the result of a former disease which had caused numerous adhesions between the two pleuræ.

Respiration is *abdominal* when the abdomen is elevated during inspiration, and depressed during expiration, while the ribs perform no motion: this phenomenon occurs when the lungs have become quite unfit for respiration; it is one of the most fatal symptoms, and generally precedes death: it is, however, often naturally abdominal in old persons whose costal cartilages become ossified by the progress of age; and, in them, the resistance of these parts is opposed to that of the already weakened muscular action.

Respiration is *thoracic* when the function is performed by the elevation of the ribs only, without the concurrence of the diaphragm: it is observed in all cases of intense extended inflammation of the abdominal organs, or in those where the abdomen is distended by pregnancy or some accidental production.

Such, says Dr. Stokes, are the changes which the motions of the chest experience in their rhythm, facility, extent, and simultaneousness. He proceeds next to treat of the pathological phenomena discovered by examining the respiratory murmur. This, he states, may be stronger or weaker than is natural, altogether inaudible, or similar to that caused by the passage of air through the wind-pipe: it may also be *cavernous*, as when the air passes into an excavation in the lung: and it is heard in combination with the different kinds of wheezing *(the râles.)* When the respiratory murmur is preternaturally strong, it bears a great similarity to that of children, and on that account has been termed the *puerile* respiration: it does not proceed from any morbid alteration of the lung at the part where it is heard, but is discerned in healthy parts whose action is, as it were, increased for a time, in order to make up for that of the diseased portions: it is met with, in one lung, when the other has lost its permeability, as from inflammation or tubercular development; in pulmonary catarrh, after the re-appearance of the murmur of respiration; and, in some cases of asthma and hysteria, but here it is combined with dyspnœa of the most distressing kind: it is heard in the sound portions, when a lung is but partially affected. We can ascertain the weakening or diminution of the respiratory murmur only by examining it at different parts of the chest; for it seldom happens that respiration is weakened in both lungs at once, or even in the entire of one: its intensity varies from the smallest diminution to the most complete nullity, and its diminution may arise from many causes. Thus, the author informs us, it is produced by the incomplete obstruction of the minute bronchial ramifications, by thickening of their membranes, or the presence of mucus; by an abundant crop of tubercles disseminated through the pulmonary tissue; by the effects of a pleurisy, while the false membranes are yet soft and only beginning to be organized; and, by the diminished action of the thorax itself.

Absence of the respiratory murmur over a more or less considerable portion of lung takes place in several of the more important thoracic lesions. Tubercles are rarely developed in such quantity as to suppress it altogether, over a large portion of the lung: when absent in *pulmonary apoplexy*, this absence is always confined to a very circumscribed space. Nullity of the respiratory murmur, indeed, seldom extends over the whole of a lung; it is almost never met with at the root of that organ, and not often at the clavicles; it is uniform and so complete in *pleurisy*, complicated with abundant effusion, that we absolutely hear nothing, except in a space of about three fingers' breadth, along the vertebral column; and even here, it is heard with less force than on the opposite side: it is a symptom, after some hours of the disease, so completely pathognomonic, that, even where the pleuritic pain does not exist, we can pronounce, without fear of error, that there is an effusion into the pleural cavity; for the disappearance of the respiratory murmur in pneumonia supervenes very gradually; and when it occurs under the clavicles, which it seldom does, this never happens until days or even weeks after the accession of the disease. When this phenomenon appears in *pneumonia*, it is always preceded by the *crepitating* wheeze, and is indicative of the second and third stages of this affection: it is not constant in *emphysema* of the lung: but, frequently, upon causing the patient to make a deep inspiration, a slight murmur is heard, attended by some of the *hissing* wheeze. Along with the clear sound obtained by percussion of the chest, this symptom forms the pathognomonic sign of a pulmonary emphysematous distention. Except in the space between the posterior edge of the scapula and the spinal column, nullity of the respiratory murmur is complete in *pneumothorax*: this, however, is not the case in emphysema: in the former, no wheezing can be discerned.

Suspension of the respiratory murmur in the affected part is one of the most remarkable phenomena exhibited by the pulmonary catarrh. This often happens suddenly, and ceases in the same manner, after some efforts of coughing or a free expectoration: it is owing to the momentary obstruction of some of the bronchial tubes by mucus sufficiently thick and abundant to intercept the passage of air, and is discontinued as soon as this obstacle has been removed. Careless observers might easily be led into error by this phenomenon, and be made to believe that the lung had become impermeable to air, or that there was an effusion into the cavity of the pleura; but it is easy to avoid this mistake; for, when the part thus affected is examined by percussion, its sound is perfectly clear, which would not be the case in pneumonia or pleurisy; and, moreover, this absence is preceded and followed by the *sonorous* and *mucous* wheezing.

Tracheal or bronchial respiration is heard in every case where the air cannot penetrate the pulmonary vesicles, as in *hepatization* of the lung, its condensation from *tubercles*, a *pleuritic effusion*, or from a *preternatural compactness* of the pulmonary tissue which, thus becoming a better conductor of sound, enables us to hear the murmur though occurring only in the large bronchial tubes. When there is a *pulmonary excavation*, the sound of breathing, as examined over it, has been termed *cavernous*: it differs from the bronchial, inasmuch as it gives the idea of air entering, from a number of small apertures, into a large and undefined cavity. This, says Dr. S. is difficult of description; but, it may be said that the sound of inspiration is more diffuse than that of expiration, in which it appears as if the air was forced through a smaller number of apertures than those by which it entered: it is a sure indication of a *cavity*, however formed. Whatever, in fine, may be the intensity of the respiratory murmur, it is either *pure*, indicating that the bronchial tubes are perfectly free, or it is *combined* with one or more kinds of wheezing. These, we are induced to describe in their order, and in this respect to deviate from the author's arrangement of exhibiting the concomitant diseases under each head, by reason of our having already given illustrations of these diseases in our preceding volumes, to which it may be sufficient if due reference is made as we proceed.

WHEEZING, *or the Râles.*—By either of these terms we are to understand any sound, produced by circulation of the air through the bronchial tubes and pulmonary vesicles, differing from the natural respiratory murmur. The varieties of wheezing seldom occupy all the lungs; but are most commonly heard over a small part only, while in other places the respiration remains unimpaired, or even becomes puerile: they indicate a narrowing of the bronchial tubes, or the presence of some liquid in them or in the air-cells of the lung: their differences of sound, their distance or vicinity, and the extent they occupy, point out the place where they occur, and some of the physical properties of the liquids giving rise to them. At a small distance from the place where they are heard, the respiratory murmur may be natural, although in the neighbourhood of a severely affected part: when a cough is present, they accompany it; but it is more convenient to examine them during respiration. These sounds may occur alone or combined with one another, either in the same or in different points: some remain through the entire period of the disease they characterise: others, which might be called intermittent, appear and disappear in turns, sometimes occupying one place, sometimes another, so that they are frequently wanting at the moment we wish to examine them. Dr. Stokes enumerates four kinds of *wheezing*; the crepitating, mucous, sonorous, and the hissing.

*Crepitating Wheeze.* This consists in a sound which has been compared to that given by salt when decrepitating, or by a piece of dry lung when pressed between the fingers. It seems to be caused by an increased determination of blood or other fluid to the air-cells of the lung, and thus forms the pathognomonic sign of pneumonia in its first stage: it also occurs, though with some modification, in œdema of the lung and pulmonary apoplexy: in recent pneumonic inflammation, it merely alters and obscures, but does not altogether conceal, the natural respiratory murmur; but, increasing with the progress of the inflammation, it arrives at its height and completely suppresses the natural sound of respiration. From its intensity, the degree of inflammation may be judged: thus, when the natural murmur predominates, the pneumonia is slight; but, when it is concealed, the inflammatory action is certainly severe and likely to terminate in hepatization of the lung. When, on the contrary, the inflammation is about to subside by resolution, the wheezing becomes distinct, emits a sound like that produced by inflation of a bladder, and acquires a more humid character; and, at the same time, the respiratory murmur grows more evident, until its complete restoration has taken place: but, when the lung is passing into hepatization, the diminution of this wheeze is not accompanied by any return of the respiratory murmur. In cases, our author adds, where the inflammation gains the superior part of the lung, the *crepitating* is frequently accompanied by the *sonorous* wheeze, indicating inflammation of the larger bronchial tubes.

In pulmonary apoplexy or hæmoptysis, this wheeze is heard in circumscribed points of the lung, more or less numerous; in the intervening spaces, respiration is heard as in health, sometimes even puerile. In course of time, this ceases to be perceived, and is succeeded by the mucous wheeze, apparently formed by large bubbles, indicating a copious extravasation of blood into the air-cells and bronchial tubes, over the whole extent of the affected part. In œdema of the lung, the wheeze which is heard has been denominated the *subcrepitating* : it is analogous to the former, but forms, as it were, a lesser degree of it, and is produced by a less tenacious fluid : it also differs from it, in being constant during the presence of the disease.

*Mucous Wheeze.* This is occasioned by the passage of air through mucus accumulated in the trachea or bronchial tubes, or through softened tuberculous matter in cavities of the lung. By its nature it denotes the viscid state of the liquid which fills the air-passages, and gives the idea of a successive formation of different-sized bubbles rising in a viscid fluid : sometimes it is obscure, and occurs at distant intervals ; in other instances, it is distinct and continued : in the first case, it shows that the collections of mucus producing it, are scattered through the lung ; in the second, that the bronchial tubes are almost filled. When it has its seat in the trachea and large branches of the bronchiæ, it can be heard without the aid of mediate or immediate auscultation : the French express the highest degree of it by the term "gargouillement," *i. e.* the gurgling sound. As this and the former kinds appear to originate from the mixture of air with liquids of different tenacity, Dr. Stokes considers them as only different degrees of the same wheeze, depending on their situation. Thus, he says after M. Andral, we hear the gurgling sound in large excavations ; the mucous wheeze, in the larger bronchial tubes ; in the smaller, a wheeze between the mucous and crepitating ; and finally, the crepitating wheeze is characteristic of inflammation in the smallest bronchial ramifications and in the air-cells of the lungs : these varieties of the same sound may be termed, they say, the cavernous, the bronchial, and the vesicular wheezes.

Mucous wheezing, in its being caused by an increased secretion from the lining membrane of the bronchiæ, is characteristic of all diseases of the lungs. It occurs in *pulmonary catarrh,* in its advanced stage ; and in *pneumonia,* in the first, where the disease is terminating by resolution ; in the second, where there is an accumulation of mucus in the large bronchial tubes ; and in the third, or suppurative stage, where an abscess is beginning to be formed. In the advanced stages of *phthisis,* where softening of the tubercles has taken place, it constitutes the proper gurgling wheeze which results from a pulmonary excavation half-filled with fluid matter and communicating with the bronchial tubes : this sound is perceived on the surface exactly corresponding to the excavation ; and, although it cannot be heard with the naked ear, yet, under the stethoscope, it is often as distinct as the rattle in the throat of a dying person. This sign, our author adds, announces the existence of an excavation in a manner almost as certain as pectoriloquism, *(pectoriloquy would, perhaps, be better, in analogy with soliloquy,)* and often appears some days before the latter becomes completely evident. When the gurgling wheeze is not very distinct, it cannot *alone* be regarded as a pathognomonic sign of the softening of tubercles, or the formation of a cavity, because partial pulmonary catarrh may sometimes present the same phenomenon ; but, when strong, and permanent in the same place, it is never equivocal.

When the tubercular mass is softened to its greatest extent, a manifest fluctuation is often heard instead of the mucous wheeze : in this case, the sound is *metallic (described afterwards,)* when the patient coughs or speaks. In some instances, this wheeze resembles the sound produced by the liquid coming from a bottle completely inverted ; but the sound is only heard momentarily, and appears to coincide with the existence of numerous anfractuous excavations, communicating with one another by canals of some length and small diameter. In pulmonary catarrh, the wheeze *first* heard is the sonorous: the mucous is not discerned till the disease has made a certain progress, when it becomes evident; still, however, permitting the respiratory murmur to be heard.

It is perceived over the affected part only, when catarrh is partial, as most frequently happens: when it extends over the whole of one lung, or a considerable portion of both, the case is always severe: when heard over the whole surface of both lungs, the disease is generally fatal, unless where the patient is very young.

*Sonorous Wheeze.* This consists of a sound sometimes extremely distinct, which resembles the snoring of a person asleep, or the tone of the bass string of a violin when struck with the finger: sometimes, also, it is similar to the cooing of a turtle, but may have a more acute character. It arises from a narrowing of the bronchial tubes, caused by determination of blood to their lining membrane, or from any other change in the form of these canals: and it may be produced by the thickening of the minute projections of their mucous coat, (called '*eperons*,' spurs, by the French anatomists) which are observed at the divisions of the larger bronchiæ; by the pressure of a tumour, or hepatization of the lung; and, not unfrequently, from mucus inspissated on the inner surface of the bronchial tubes.

With his characteristic precision, the author cautions us against confounding this kind of wheezing with the *guttural* sound produced during sleep: the first, he says, has its seat in the chest, and is not heard by the naked ear; the second, on the contrary, is solely derived from the manner in which the air, inspired and expired, strikes the velum of the palate. by means of the stethoscope, it is easy to discover that it does not take place in the cavity of the chest. The sonorous wheeze is the pathognomonic symptom of *acute bronchitis:* it is complicated with the crepitating, in *pneumonia* accompanied with bronchitis; and with the hissing in asthma, or the dry *pulmonary catarrh:* the first varies little; the second, or hissing, is very fitful, disappearing for an uncertain space, in consequence of coughing, or without any perceptible cause, and then returning suddenly and with great intensity: sometimes both are constant, distinct, and perceivable, over the greatest part of the organ; in which case, the affection is extensive and violent. In the *humid catarrh,* the same phenomena may exist; but, ordinarily, they are combined with the mucous wheeze, which becomes entirely predominant when the acute stage is past, and thus characterises the disease.

*Hissing Wheeze.* This is a prolonged wheezing sound, accompanying either the end or commencement of inspiration or expiration. It may be grave or acute, dull or shrill; and these varieties occur in different parts of the chest, or meet together in the same point, at greater or less intervals. Sometimes it is of very short duration, and has been compared to the cry of young birds, to the sound caused by two pieces of oiled marble, when suddenly separated, or to that proceeding from the action of a small valve: it is owing to the presence of viscid mucus, obstructing the lesser bronchial ramifications, through which the air is obliged to pass before reaching the vesicles. Respiration is very laborious when this wheeze is heard over a considerable portion of the lung; and it is during its existence that we observe the sputa presenting an arborescent appearance, resembling the form, calibre, and branches, of the minute bronchial tubes, from which they have been expelled by coughing. It is connected principally with

*emphysema* of the lungs and the *chronic pituitous catarrh:* it is combined, in the *acute,* with the sonorous and mucous kinds. In emphysema, says Dr. S. the respiration is not heard over the affected part, while the chest sounds well, or even louder than is natural, on percussion: a slight hissing sound only is heard, from time to time, at the points of the surface corresponding to the disease's site.

Following his description of the mucous wheeze, at p. 37, Dr. Stokes has a good section on *pneumonia,* both acute and chronic, with two cases in illustration: as a reasonable degree of attention, however, was paid to the subject in our Number for January, 1820, p. 474, and that for October, 1825, p. 508, there does not seem to be any necessity for our resuming it: what our author here advances on this topic is precise and practical, and we recommend it earnestly to the consideration of commencing auscultators. At p. 483, of the same Number, we described *Œdema of the Lungs,* with its pathology, which leads us to pass on, with merely mentioning, that Dr. Stokes' account of the same affection is faithful and perspicuous, and the case he has selected, as an example of it, is particularly appropriate. Had we not already, ib. p. 485, drawn a detailed picture of *Pulmonary Apoplexy,* we should have been tempted to transfer that so ably delineated by Dr. S. p. 60, to the pages of this Journal: it is one of the clearest pathological sketches we ever had occasion to examine. In our last Number, p. 496, we gave a pretty full view of *Bronchitis*: Dr. S. has, with great judgment, at p. 70, adopted M. Andral's pathology of this insidious complaint: his own remarks, with reference to it, are made with much discrimination: the reader will be pleased and instructed by a comparison of the two descriptions. Under the same head as bronchitis *(Pulmonary Catarrh,* p. 69, 77,) he explains concisely the pathological phenomena of the organic change in the air-tubes, which Professor Laënnec originally denominated *"dilatation of the bronchial tubes."* Some idea may be formed of the importance we attach to this subject, from the consideration of our having submitted it, on three former occasions (*January,* 1820, p. 470, *September,* 1824, p. 451, *and October,* 1825, pp. 500, 502,) to our readers' observation: it is a subject, indeed, which has not yet been fully investigated; and, for this reason, we press it on the attention of every pathological inquirer.

Although very much satisfied with the brevity and truth wherewith Dr. S. has illustrated the pathology of *pulmonary emphysema,* p. 79, we cannot do more at present, than advise all young practitioners to study it carefully, and at the same time to call to their assistance what we have introduced, in our Number for January, 1820, p. 476, for the purpose of elucidating this disease, whereof the symptoms are in many instances equivocal. This brings us to our author's chapter on the *phenomena of the voice,* which consistently with his plan, he considers under their natural and pathological relations. To what we said on one branch *(pectoriloquy)* of this subject, in a former article *(January,* 1820, p. 462) we shall now add a general outline of Dr. Stokes' observations.

*Natural Phenomena.*—These vary, 1st, according to the points examined, and 2d, according to the tone of the voice. When a healthy man speaks or sings, his voice resounds in the inside of the chest, and produces through its whole extent, a sort of trembling or vibration easily distinguished by applying the hand. By means of the stethoscope, while the individual is speaking, a confused resounding of the voice is perceived, and the intensity of this varies in different points of the chest: it is most distinct in the arm-pit, on the back between the internal border of the scapula and the spinal column, and in the angle formed by the union of the sternum and clavicle. In these points, the voice seems stronger, and as if it were closer to the observer's ear: in the rest of the chest, particularly the inferior and posterior regions, it appears weaker and more distant, and only produces a confused sound in which nothing articulate can be distinguished. In men, whose voices are deep, the resounding is stronger, but dull, confused, and almost of equal intensity in all points; while, in women and children, it is clear and very distinct.

*Pathological Phenomena.*—These are of four kinds; bronchophony, pectoriloquy, metallic tinkling and egophony, which we shall sketch in succession.

*Bronchophony.*—This is a vibrating sound of the voice, louder than natural, or occurring in a point where it is not heard in the state of health: it is an inarticulate sound or confused noise, which barely seems to enter the bottom of the stethoscope, without traversing the tube to arrive at the ear. Induration of the pulmonary tissue produced by inflammation or by a mass of crude tubercles, becomes its cause, by rendering the lung more fit for transmitting the vocal murmur. An accurate idea of this symptom is obtained by applying the instrument on the point of the chest corresponding to the root of the lung, while the individual is speaking: when it occurs in extensive pulmonary hepatization, it is always accompanied by the bronchial or tracheal respiration. Sometimes, it is of great importance, by enabling us to institute a comparison between the two sides of the chest; and, from its co-existence with other signs ascertained by different modes of examination, leading to a more certain conclusion.

*Pectoriloquy.*—Patients are pectoriloquent when the voice, distinctly articulate, seems to issue directly from the place where the stethoscope is applied, and to traverse the canal of that instrument. The symptom is either perfect, imperfect, or doubtful: it is *perfect,* when the articulate and well defined voice traverses the cylinder, and arrives at the ear with its natural, or an increased intensity of sound; *imperfect,* when the articulate voice reverberates strongly under the stethoscope, appearing to approach the ear without traversing the entire tube; and *doubtful,* when the voice appears sharp and restrained, like that of those who practise ventriloquy, and does not pervade the tube as in simple bronchophony: the two last can only be trusted to, as indicative of organic lesion, when they exist in one side solely, or when they co-exist with other symptoms observed by examining the respiration. For a short time, the most perfect may sometimes take on the character of the imperfect, or even the doubtful kind; and it may disappear from time to time, and thus become intermittent.

Pectoriloquy is owing to excavations in the lung, either partially or completely empty, and communicating freely with the bronchial tubes: it may occur in all parts of the chest, but most frequently in those which correspond to the superior portion of the lung, where the excavations produced by the softening of tubercles are oftenest discovered: and it varies with the sound of the voice, the size of the excavations, their form and the density of their sides, the adhesion of the two pleuræ over these cavities, and the facility or difficulty with which the air enters them. It is evident in proportion to the acuteness of the voice: in persons with a deep voice, it is almost always imperfect, sometimes doubtful. Aphony does not cause it to disappear completely, and it often happens, that what the patient says, can be distinguished better by the stethoscope applied over the excavation, than with the naked ear at the same distance.

This phenomenon can be perfect only when the excavations are of a moderate size: when these are large, it is changed into a deep sound, analagous to that of the voice transmitted to some distance through a trumpet or cone of paper; but, where they are small, it is often doubtful, especially if the cavity be situated in the centre of the lung, and surrounded by parts still easily permeable to air. It appears somewhat stifled and confused, and the voice is badly articulated, when there is irregularity, or the direct communication of a number of cavities with one another; and, the firmer and thinner the sides of the excavations are, the more perfect it is. When, says Dr. S. by a process of cicatrization, a fibro-cartilaginous membrane is formed over the entire surface of one of such cavities, the pectoriloquy acquires a metallic tone, sometimes so considerable as to hinder our accurate perception of the sounds. It is not caused by excavations situated at the surface of the lung, and whose thin sides do not adhere to the costal pleura, but collapse during expiration; but it is so strong as to offend the ear when occasioned by a superficial cavity with thin *adherent* sides: besides, it is more evident in proportion as the excavation contains less fluid, because the bronchial communication is then generally free, and permits an easy access to the air. This communication, however, may be obstructed more or less completely by the accumulation of sputa in the bronchial passages: this renders the *perfect* pectoriloquy *doubtful*, and gives it that intermittent character which not unfrequently supervenes. When this symptom is absent in a patient who had it on the evening before, it may often be remarked, that the expectoration has been scanty, or almost entirely wanting. Dr. Stokes exemplifies these doctrines by two cases accompanied with dissections: the first is from Laënnec, the second from Andral; and both are most instructive.

*Metallic Sound, or Tinkling.*—The remarkable sound so denominated is of short duration, and takes place, upon raising the patient or making him cough: it is analogous to that produced by a drop of water falling into a deep vessel, a grain of sand into a glass cup, or to that caused by striking a metallic or porcelain vessel with a pin: it seems as if a drop of fluid detached itself from the upper part of a cavity and, by falling into the mass of liquid at the bottom, occasioned by its shock this peculiar sound. It is heard when the person breathes, speaks, or coughs, but much more distinctly in the two last, than in the first of these actions: there are some exceptions, however to this general rule; but coughing renders it so very evident that it is advisable to make the patient cough in order to be assured of its existence. When it co-exists with pectoriloquy, it is perceived traversing the stethoscope along with the voice; but, if the latter does not accompany it and the phenomenon is produced by the voice, a slight acute sound is heard vibrating in the chest, analagous to that determined by striking a metallic chord with the finger.

As this peculiar sound, our author observes, depends on the vibration of air, caused by respiration, the voice or coughing, on the surface of a liquid partly filling an unnatural cavity in the chest, it can only exist in two cases; 1st, where a serous or purulent effusion co-exists with pneumothorax, arising from a fistulous opening into the cavity of the pleura; and 2d, where a large excavation, half filled with fluid pus, occurs in the substance of the lung. It is necessary to its happening in the first case, that there be a fistulous communication between the pleural cavity and some of the bronchial tubes; thus it becomes a sign of this triple lesion. The distinctness of the sound is in proportion to the diameter of the fistulous opening, and the extent of the vibrations indicate the space occupied by the air: it is in general stronger as the quantity of air in the chest is greater; hence it has been concluded, that, when it is indistinct, the liquid effusion is considerable, and *vice versâ*. When, in fine, it arises from the vibrations of the voice, or from coughing, acting on the surface of puriform matter in a large excavation of the lung, it presents some important differences: its indistinctness and the small extent of its vibrations shew that it occurs in a very circumscribed space; it appears to enter the cylinder and is combined with pectoriloquy which, with the other symptoms, enables us easily to distinguish *this* from the former case.

*Egophony.*—This denotes a strong reverberation of the voice, which seems more acute than that of the patient: it is shrill, interrupted, and quivering, like that of a goat, the tone of whose voice it much resembles. It may be produced over the whole extent of the chest, on one side only or both at once: most frequently it occurs within a circumscribed space, the limits of which are formed by the vertebral column and the internal edge of the scapula: it is also found at the inferior angle of this bone, and in a space three fingers in width which, following the direction of the ribs, passes from the middle of the scapula to the sternum. It varies much in its force and extent; is heard over a much greater space than pectoriloquy; and always appears to indicate the existence of a small quantity of liquid in the cavity of the pleurâ, or the occurrence of thick pseudo-membranes in a soft state. It ceases to exist when the effusion becomes very abundant or is greatly diminished; and it is not heard when the cavity is suddenly filled in this manner.

Egophony is owing to the natural reverberation of the voice in the bronchial tubes, transmitted through the medium of a thin and trembling layer of effused fluid, and made more evident by the compression of the pulmonary tissue, which is consequently better adapted for the transmission of sound. Many facts support this opinion, thus, the points where the egophony is most frequently heard, are those which indicate the superior part of the effusion, and, of course, its thinnest portion: this has place when the patient is lying on his back or seated upright. When, on the contrary, he lies on his belly, it is either not heard at all, or at least very feebly, in the space between the scapula and spinal column, while it continues to be perceived at the side: it also becomes less apparent, when he lies on the side opposite to that which is affected. Another circumstance goes to confirm the sentiment, *viz.* the cessation of egophony when the effusion is very abundant, and its return when this abundance diminishes; for, when the effusion is very considerable, the bronchial tubes themselves get to be compressed like the pulmonary tissue; and, when it is diminished, they necessarily resume their volume before the latter, on account of their greater elasticity.

Egophony is rather a favourable sign in pleurisy, inasmuch as it indicates that the effusion is not very great. When several circumscribed pleurisies exist, it occurs at the same time; and, here, says Dr. S. the usefulness of the stethoscope must strike the most ignorant or prejudiced observer, as it points out the seat of the disease with the utmost certainty, and directs us in the application of our most powerful topical remedies. This word, in fine, is to be regarded only as a *generic* term, under which various modifications of the quivering sound, not resembling the voice of a goat, are comprehended. Frequently, these different degrees of the egophonic sound occur merely at intervals, or are heard in the pronunciation of certain words: hence, it may happen that these numerous varieties shall lead to frequent illusions, and that it shall even be possible to regard as a pathological phenomenon what, in truth, belongs to a state of health. This error, however, may be avoided, by our never pronouncing whether egophony exists or not, until we have heard the voice on the side presumed to be healthy: it has often occurred, indeed, that physicians, after having believed that this and other signs indicative of effusion were present, have discovered their error by examining the opposite side.

Passing the section on *Phthisis*, p. 94, the stethoscopic pathology of which we considered at some length in the Number of this Journal for January, 1820, p. 463, 466, and that on *Pleurisy*, both acute and chronic, to which we rather particularly directed the reader's attention at pp. 479, 481, of the same Number and again in March, 1822, pp. 796-801, we may just add as we proceed, that Dr. Stokes has concentrated much information on the subject in a manner distinguished alike for perspicuity and elegance.—We are now arrived at the Doctor's short, but very practical section on the

Measurement of the Thorax. By this process, the development of one side of the chest is compared with that of the other, and the comparative contraction or dilatation of either ascertained. Dilatation takes place in cases of chronic pleurisy, where there is no tendency to the absorption of effused fluid: in these, the affected side appears to the sight much larger than the opposite one; the chest seems inflated; the intercostal spaces are on a level with the ribs; and the sound, on percussion, is completely dull. This state occurs in empyema, pneumo-thorax, and emphysema; but, in the two last, the percussive sound is increased. As many persons have the right side naturally a little more developed than the left; and others, from the effects of early rachitis, present this peculiar conformation or perhaps a contraction of the chest, the physician must not trust to measurement alone as a means of diagnosis: these should make him inquire accurately into the patient's previous history.

When the thorax, divested of all clothing, is being examined by measurement, the person should be made to sit upright, with his arms in a dependent posture or joined over the head; but they must retain always the same relative position, for the state of muscular relaxation or contraction causes an evident difference in the dimensions. By the operator standing behind, the semi-circumference of the chest is to be taken by a tape, from the spinous processes to the middle of the sternum; and then, turning the tape without moving its extremity from the spinous process, we are to measure the opposite side in the same manner.

We have now travelled with Dr. S. over the first grand division of his Essay, and should now have much gratification in accepting his guidance through the second, in which he discusses the natural and pathological phenomena of the heart. Under the first of these heads, he considers the extent of the pulsations of the heart, their stroke or impulse, the sound produced by them, and their rhythm or the order in which the different parts contract and the respective duration of these contractions to each other: under the second, or pathological, he includes those phenomena which relate to the extent in which the pulsations of the heart can be perceived, to the force of its pulsation, and to the nature and intensity of the sound thus produced. His sections, which are remarkably instructive, embrace the following subjects, illustrated by cases; viz. dilatation of the heart; dilatation with hypertrophy; hypertrophy; structural softening; pericarditis; disease of the cardiac valves, and aortal aneurism. In our article on Professor Laënnec's "immortal work," we gave as complete a view of the pathology of the heart (Number for January, 1820, p. 489,) as could reasonably be expected within the compass of an analytical sketch: to that article, therefore, we simply refer; but, in doing this, we conceive it to be our duty also, as well to the zealous author as to our readers, to urge these discussions on the deep and frequent attention of all, especially those who are inexperienced in the practice of stethoscopy.

Dr. S. has appended to his work two very interesting sketches: one on gangrene of the lung, and one in the form of an abridged translation of M. Andral's Thesis, 1821, on expectoration. In our last Number (October, 1825) p. 521, we gave a case illustrative of this rare termination of disease: to this, therefore, and to another adduced by our author we merely refer; and, in the mean time, shall attempt from his pages a brief sketch of the affection. Whether its character be acute or chronic, pneumonic inflammation may, though rarely, terminate in gangrene. When such does occur, it usually appears under two forms: in the first, it is circumscribed; in the second, it affects a more or less considerable portion of the lung. *Circumscribed* gangrene is described as giving rise to an excavation in the pulmonary tissue, and being without a distinct lining membrane like the tubercular cavity: it may occupy the surface of the lung, but is most commonly found in the interior of this organ. When not complicated with other affections, the ulceration may occur in the centre of a lobe, while around it the pulmonary tissue is healthy and crepitating: it almost always exhales a very fetid and gangrenous odour; and its surface, which is very irregular, is generally covered with a greyish-brown, or even black purulent matter: its extent is variable; sometimes, it may contain a nut; in other instances, it is capable of holding three hens' eggs.

Pulmonary gangrene of this kind, admits of three stages, that of recent mortification, that of deliquescent sphacelus, and that of excavation formed by evacuation of the gangrened part. In some cases, the sides of this cavity are lined with a soft, opaque false membrane, having a greyish hue, secreting pus of the same colour, and emitting the gangrenous odour; but this membrane frequently does not occur, and the walls of the ulcer are then dense, greyish-brown and, when cut, present a granulated aspect. This state of induration is produced by the third degree of inflammation of the lung, and generally extends to about an inch from the excavation; but, it has also been seen to engage the whole of a lobe. in other instances, the sides of the cavity are soft and putrescent: isolated and denuded blood-vessels sometimes traverse the excavations; but they have also been found destroyed, while their mouths opening into the hollow, have filled it with a clot of blood.

In *uncircumscribed* gangrene of the lung, the pulmonary tissue is moister and more friable, than in the natural state: its density is the same as in the first degree of pneumonia, œdema of the lung, or in the sanguineous infiltration which occurs in dead bodies: and its colour varies from a greenish-white to a dark green, sometimes with a mixture of brown or brownish-yellow. These shades appear in different parts of the lung; and we may also distinguish more humid portions of a livid-red, which seem infiltrated with very fluid blood, exactly similar to the lung in the first stage of inflammation: here and there, points are observed in a state of putrid softening, while a sanious greenish liquid having an insupportable fœtor, flows from the surfaces of the incisions. This lesion may occupy nearly the whole of a lung: in some parts, the healthy pulmonary tissue passes insensibly into the gangrened portions; but it may be separated from this by a portion of lung inflamed only to the first degree: this separating portion is sometimes, though rarely, partially hepatized.

Our limits admonish us to be brief; but still we feel desirous of attempting to abridge Dr. S's. excellent abridgement of M. Andral's Thesis on expectoration: let us try.—Expectorable matter, then, is furnished by many sources, but most commonly from the bronchial mucous membrane; but this, subjected, like all exhalent surfaces, to numerous different causes of irritation, can secrete fluids which vary infinitely with regard to their physical character and chemical composition: thus, in pneumonia, the secretion is a viscid mucus, transparent and bloody; in acute catarrh, it is limpid and colourless; in the chronic, opaque and puriform; and, in croup, it concretes into false membrane. With these, also, are mingled in varying quantities, the liquid furnished by the tonsillary and other oral glands, and the mucus of the pharynx. Sometimes, the expectoration is pure blood; in other cases, it is composed, partially or totally, of accidental productions, *e. g.* tubercles, calculi, melanotic matter, hydatids, developed in the pulmonary structure which they have more or less altered: in some instances, in fine, it contains purulent matter formed originally in the cavity of the pleura, in the mediastinum, and more rarely in the liver.

In many cases, it is possible to pronounce what is the state of the lungs by examining the expectoration: thus, in pneumonia, we can not only detect the disease's nature by this means, but even trace its different stages with sufficient precision: in phthisis pulmonalis, however, this is difficult or unattainable. Consideration of its nature, moreover, not only aids the diagnosis, but even affords many useful hints on the disease's progress, its duration, and termination. Occasionally, it is critical, and many affections resolve themselves by a salutary expectoration: now and then, it is so sudden and profuse as to destroy the patient by suffocation.

Expectoration in Pulmonary Catarrh.—At first, the cough is dry, and its continuance in this state marks the disease's first period: by-and-by, this is followed by the expectoration of a transparent glairy mucus, which is very viscid and sometimes thready. Its tenacity is proportionate to the violence of the inflammation, and this viscidity is usually concomitant with the febrile paroxysms: when the sputa are suppressed during these, great irritation of the mucous membrane is indicated. Sometimes, at the close of the diaphoresis which terminates the febrile fit, a copious, thick, opaque expectoration supervenes; but this is only temporary, and turns to clear mucus as the accession of fever approaches.

Generally, the quantity of froth on the surface of the sputa depends on the facility with which they are rejected: they are combined with a great quantity of air, forming a froth which is very difficult of separation, when the patient expectorates only after a long fit of coughing, during which the air, often inspired and expired, is intimately mixed with the mucus filling the air-passages. Early in catarrh, they are often streaked with blood from the rupture of small vessels by the efforts of coughing: the blood, however, is only *mixed* with the mucus, not *combined* with it. Occasionally, also, small grains of a whitish colour are found in the expectoration; but these do not come from the lung; they are secreted by the pharyngeal and contiguous glands, and should not be mistaken for particles of pulmonary tubercles.

So soon as the inflammation is about to terminate in resolution, the sputa change their character, lose their transparency, and become mixed with yellowish-white or greenish opaque masses which, gradually increasing, form at length the entire expectoration and announce that the inflammation is resolved: towards the conclusion of acute catarrh, their characters are very variable; but, when the disease passes into the chronic state, they are opaque, and whitish-yellow or greenish, sometimes adhering to the bottom of the vessel, sometimes suspended in transparent mucus. Most frequently, they are inodorous, insipid, easily rejected, and most abundant in the morning: when the complaint is protracted, they differ little from what has place in the acute stage: in such a case, if the expectoration is copious, hectic fever and death may be the result: some sink rapidly under it, having phthisical symptoms; others die of exhaustion before a well-characterised hectic is established; while some support enormous discharges, without much constitutional affection.

Among the many causes of asthma is excessive secretion from the bronchial membrane; and persons in whom it prevails, usually have a slightly laborious respiration: when they cease to expectorate freely, or if the secretion be suddenly increased, they immediately experience a sense of suffocation; but, upon removal of the cause, they soon recover. In chronic catarrh, the expectoration sometimes occurs periodically: and several female cases are on record, of its being purulent, fœtid, and copious, at the time of each menstrual return: at other times, it is abundant and muco-puriform at the close of a disease, and then appears to be critical. Long-established expectorations and the discharges from certain old ulcers are to be looked upon as natural evacuations and, in consequence, not rashly interrupted.

Every acute or chronic disease of the pleura and lungs, most of the organic affections of the heart, many disorders of the stomach and liver, and some of the skin, do more or less affect the bronchial membrane; hence the complications of pulmonary catarrh: but then, the secretion varies according to each different irritation. According to this view, the frequent and distressing cough in gastritis is generally dry, but it may be accompanied by an expectoration modified by the degree and duration of the bronchial irritation. Yellowness of the sputa is one of the characteristic signs of hepatic inflammation; but, when cough attends this disease, it is dry. They also have a distinctly yellowish colour in many bilious fevers; but in jaundice it is very variable: sometimes they are white, as is the tongue, while all the other liquids of the body are of a deep yellow; at other times they are bright yellow, and the tongue has then the same hue: in some very rare instances, they are so green as to have the appearance of being impregnated with the resinous matter of the bile, and then the thick crust covering the tongue has a similar colour. In the catarrh of measles, they have a great analogy, especially at the close of the disease, to that in consumption: occasionally, in the advanced stage of adynamic fever, persons expectorate a small quantity of thick, viscid, ash-grey matter, resembling the purulent discharges of phthisical patients a short time before death: nevertheless, in such cases, there is no lesion of the pulmonary tissue. Dilatation of the bronchial tubes in fine, is frequently distinguished by cough and a muco-puriform expectoration.

Expectoration in Pneumonia. This, generally, is transparent, tinged with blood, uniting into a gelatinous mass, and so viscid that the vessel containing it may be inverted without its being detached from the sides. When, at the disease's commencement, there is already cough, dyspnœa, fever, and a deep-seated pain in the breast, the patient only expectorates a little bronchial mucus mixed with saliva: still, however, the chest gives a clear sound on percussion, but the crepitating wheeze begins to be heard on one side, while, on the other, the respiratory murmur is stronger than in the natural state: and, according as the wheeze becomes more evident, the expectoration gets to be more distinctive, which usually happens on the second or third day. When tinged with blood, it is either yellow, or of a ferruginous colour, or of a bright red, in proportion to the quality of this fluid: at the same time, it becomes viscid and forms a transparent and homogeneous mass; but, on inclining the vessel wherein it is contained, it still easily flows out: thus, at this early period, the expectorated substances adhere strongly together, but have not enough of viscidity to adhere to the sides of the containing vessel. When, however, the pneumonia has made progress and passes into the second degree, they acquire a much greater viscidity, and no longer detach themselves from the vessel; the chest sounds dull on percussion, and the respiratory murmur can be no longer heard; the inflammation reaches its highest intensity; and the expectoration remains for some time stationary. According to the termination of the disease, it proceeds to exhibit new characters; thus, if resolution is about to take place, its viscidity and sanguinolence diminish: at first, a little agitation is required to separate it from the vessel; somewhat later, a slight inclination will suffice; by degrees, it returns to its pristine state; and, at length, is similar to that in common acute catarrh: its again becoming viscid and sanguinolent proves a certain exacerbation; and, when this supervenes, all the other symptoms increase in severity. Nevertheless, the disease may terminate favourably, although the sputa, which have lost their viscidity, and are no longer tinged with blood, remain aqueous, transparent, and colourless; and, in the end, cease to be expectorated, without having acquired a greater degree of *coction*. Though they have become purely catarrhal, however, we must not conclude that perfect resolution has taken place; because it often happens that the expectoration, with all the other symptoms, shall indicate a complete resolution, while the dullness of sound and crepitating wheeze still continue: these last signs occasionally survive all the rest, for 10, 12, or 15 days. The true pneumonic expectoration less frequently continues after the cessation, or, at least, the amelioration of the other symptoms. Great viscidity of the sputa, at the outset, is always an unfavourable circumstance, and tends to retard or prevent resolution, in consequence of its making the expectoration difficult, increases the dyspnœa, and aggravates the disease.

When the pneumonia advances to suppuration, the sputal excretion, in a majority of patients, becomes difficult and scanty, and at last altogether ceases; but it generally happens, that the secretion of expectorable matter continues, while its excretion is impossible, by reason of the patient's debility: hence, it accumulates in the bronchial tubes, trachea, and larynx, obstructs these passages, and occasions death from asphyxia, as is proved by the strong tracheal wheeze which is then heard. In other cases, the secretion of expectorable matter ceases with more or less rapidity; and then the state of the bronchial membrane may be compared to that of wounds whose surfaces, after long suppuration, have suddenly become dry. Among the many causes which thus diminish or suspend the bronchial secretion, may be reckoned the numerous affections with which pneumonia is so often complicated. According to Baglivi, the exhibition of much purgative medicine at the disease's commencement, suppresses expectoration and is consequently hurtful: Morgagni regarded untimely bleedings, especially in old persons, as productive of the same effect: and Sydenham declares that bleedings too often repeated will suppress this secretion; but when performed with judgment, will often re-establish it: detraction of blood, to the proper extent, is undoubtedly one of the best expectorants. When suppressed expectoration arises from retention of the sputa in the air-passages, the case is always severe; but, when from interruption of the bronchial secretion, there may be error in a prognosis formed without consideration of all the other symptoms, patients labouring under the most intense pneumonia, sometimes suddenly cease to expectorate, without sustaining any injury; but, in them, the recovery is generally very slow. Some authors have stated, that another excretion may be established at the time when expectoration is suppressed; but this is unfrequent. In some persons, threatened with instant death, the sputa continue, but are changed in their aspect: during the last 24 hours of their existence, many of such patients expectorate a small quantity of opaque matter, having a dirty reddish-grey colour, and collected into masses which possess great affinity to those discharged by phthisical persons immediately before dissolution. In a few rare instances, the sputa continue until death, with the same characters and in the same abundance as if the pneumonia were proceeding to resolution.

In fatal cases, when the lung was found infiltrated with pus, some patients had ceased to expectorate: in others, the sputa had an opaque reddish appearance: but, in the majority, they had lost their gelatinous nature, viscidity, and reddish colour, and seemed to be formed of a brownish, sometimes black liquid, bearing a strong resemblance to the juice of prunes. Nevertheless, such appearances occur in the sputa of patients, whose lungs were afterwards found in the state of red hepatization: even in slight cases of pneumonia, this has been observed. By these anomalies we are taught, that inspection of the sputa can only furnish us with *probable conjectures*, but never with *absolutely certain information* as to the progress of the disease or its eventual termination.

Primitive chronic pneumonia is attended by an expectoration which presents all the different shades that occur in pulmonary catarrh. If the disease assumes an acute form for a time, the change is announced by the expectoration again becoming viscid, transparent, and tinged with blood. When inflammation supervenes on any former disease of the lung, the original sputa disappear altogether, and are replaced by the pneumonic; but frequently there is a mixture of both, and then, no conclusion regarding the prognosis or diagnosis can be drawn. At other times, after the pneumonic has appeared for some time, the former expectoration returns at the decline of the disease, and thus occasions a new source of error. In such cases, the white and opaque sputa have been considered as critical, while, in truth, they were the product of an old bronchial affection, suspended or modified by the pneumonia, and re-appearing as soon as this last began to be resolved. Pneumonia without expectoration occurs chiefly in severe fevers complicated with inflammation of the lung; in these, the debilitated patients, often deprived of their intellectual faculties, generally swallow the sputa, not having strength or instinct to expectorate: occasionally, however, no sputal matters are secreted. In pneumonia, these are evidently furnished by the bronchial membrane, sympathetically irritated by the pulmonary inflammation: as this subsides, that of the bronchial membrane loses its intensity and the sputa become those of simple catarrh. According to the different degrees of bronchial irritation, expectoration does not take place as the secretion of sputa is suppressed: the prune-juice-like sputa are connected with a peculiar inflammatory state of the bronchial membrane; but, in this, as in other inflamed tissues, the secretion varies according to the degree or the character of the inflammation.

Expectoration in Pleuritis. When this kind of inflammation is acute, there are generally no sputa; but, if these do appear, they are similar to those of acute catarrh, *i. e.* abundant and sanguinolent: they are not different when pleuritis terminates in effusion; but, if a communication is established between the cavity of the pleura and the bronchial tubes, the effused liquid is evacuated through the trachea, and is found in the matter of expectoration. It has been said that the purulent matter secreted in the pleura differs altogether by its physical characters from the bronchial mucus or the liquid secreted in phthisical excavations; but these distinctions are difficult of verification at the patient's bed-side. The extreme fœtor of the sputa and their alliaceous odour are often set down as sure indications of their pleuritic origin: but, it may be asked, is not the expectoration of consumptive persons often as fœtid? It is a fact, moreover, that there have been persons whose sputa were inodorous, in whom, nevertheless, a communication *did* exist between the cavity of the pleura and the bronchial tubes. In regard to its colour, consistence, and form, the expectoration of pleurisy is not dissimilar from that of chronic catarrh. When, in fine, a pleuritic effusion is freely evacuated through the lungs, this evacuation takes place suddenly, and the patient seems to vomit purulent matter: but, although this may be the case when a large opening is formed, it is evident that the expectoration will be gradual when the aperture is small.

Expectoration in Pulmonary Emphysema. This state of the lung is generally accompanied by the symptoms of catarrh, and the expectorated matter is very variable in its character: the cough is sometimes dry, sometimes followed by the expulsion of a greyish, transparent, and more or less viscid fluid: at other times, the expectoration is thick and opaque.

Expectoration in Pulmonary Œdema. Like that of acute catarrh the expectoration in this disease is aqueous and transparent; but, as œdema of the lung is rarely a primitive affection and may occur in many diseases where the bronchial membrane is already affected, such as chronic catarrh, pneumonia passing into resolution, lesions of the heart and various fevers, it is probable that the expectoration may exhibit a great diversity of aspects.

Dr. S. refers his readers to the cases of gangrene of the lung related in his Appendix, for a description of the expectoration in this uncommon malady: he also omits the characters of expectorated matter as drawn from the use of chemical tests, conceiving, with M. Andral, that they are not determined as yet with sufficient accuracy.

Expectoration in Phthisis. It is by the consideration of their *physical* properties that the sputa, peculiar to the tubercular degeneration of the lungs, can be best recognised. In commencing phthisis, when some lesion of the pulmonary organs more important than simple catarrh, is indicated by cough with frequent hæmoptysis, emaciation, and irregular febrile attacks, the sputa are yet without character. In many, the cough is dry: in others, it is accompanied by a purely catarrhal expectoration which, although of long duration, still retains its original appearances. This circumstance should never be overlooked: it always affords reason for suspecting the existence of tubercles. After this dubious catarrh has remained for a long time, if the sputa be examined daily, small yellowish-white grains will be observed in them: these particles have a moderate consistence, and vary from the size of a pin's head to that of a pea: they remain separate, and fall to the bottom of the vessel; and, when broken, exhale a very fœtid odour which has been regarded as pathognomonic of pulmonary consumption. These granular bodies, however, must not be confounded with those secreted by the pharyngeal glands, which are exceedingly viscid and tenacious, and present a strong contrast to the friable tubercular granules. Dr. Stokes proposes an ingenious and simple method of distinguishing these substances by heating them on paper: the secretion of the tonsils and neighbouring glands is sebaceous, and consequently greases the paper; but this is not the case with tubercular matter.

During the first period of the disease, the sputa are sometimes composed of a transparent colourless liquid, wherein are suspended long and delicate threads which occasionally float on the surface of an opaque mucus, and are distinguishable by their dull white colour. If death takes place, in this stage, the lungs are studded with small tubercles, some of which are hard, and others beginning to soften towards their centre: at this time, it is rare to meet with tubercles completely softened, or with excavations of any size. Persons having long had a dry cough with all the other symptoms of crude tubercles in the lungs, sometimes suddenly expectorate a large quantity of puriform matter, coming from a tuberculous excavation communicating with one of the bronchial tubes,—a state that may prove fatal, but from which some persons have recovered. As the above-mentioned grains and delicate filaments become more abundant, they unite into differently sized masses which remain suspended in a clouded serosity and form what has been called the *flocculent sputa;* in other instances, they are round and thickened, and perfectly separate from one another; these are the *nummular sputa*, the appearance of which forms one of the most fatal symptoms. When examined with the naked eye, they are seen to be formed by a multitude of little points capable of more minute division: these whitish molecules are united by a semi-transparent greyish mucus, which sometimes, however, is yellow or greenish, and completely opaque; hence the expectoration has a variegated appearance: on other occasions, the sputa are composed of long delicate filaments sometimes twisted on themselves, and united by mucus from which they are distinguished by their colour. At this period, indeed, the expectoration presents innumerable shades of difference which depend on the manner of communication of the bronchial, with the tubercular excavation; on the number, length, size, and mode of division of the bronchial tubes through which the matter must pass before coming to the wind-pipe; on the quantity and nature of the mucus with which it is mixed; and, on the length of time it has remained in the bronchial tubes before its expulsion. These varieties, however formed, constitute the *compound sputa*, and are certainly indicative of tubercles in a complete state of softening, with deep excavations of the pulmonary tissue.

During the latter periods of phthisis, a dirty ash-grey liquid is secreted from the sides of the tuberculous excavations: sometimes it has a reddish colour, which arises from the mixture of a certain quantity of blood: and sometimes it has a great analogy to the sanious pus of old and ill conditioned ulcers, and is frequently mixed with small grains of decomposed tuberculous matter. When the excavations contain this fluid, its existence is generally revealed by the expectoration, in which it occurs at first in small quantities, but gradually increases so as, at last, to constitute nearly the entire mass: it is then almost a homogeneous pus, sometimes fœtid, sometimes inodorous, and contains particles of softened tuberculous matter: these particles, however, do not always occur; for there have been cases where, although found in the excavations, they could not be detected in the expectoration. If the sputa still continue divided and do not take on the puriform aspect, it often happens that, 24 or 48 hours before death, their character is altogether changed, the serosity disappears, and the rejected matter forms a thick greyish mass, strongly adherent to the bottom of the vessel. In other cases, expectoration is totally suppressed a short time before death: the symptoms are then aggravated, and the strength rapidly declines: this, indeed, is justly held to be a very fatal symptom. As in pneumonia, it may arise from two causes,—from the inability of the patient to expectorate, when the sputa accumulate in the wind-pipe, and he sinks asphyxiated; and from the expectoration being suddenly suppressed without any tracheal wheeze having been heard, while at the same time the mucous wheeze, which indicates a cavity filled with fluid, ceases to be observed, although a short space previously, it had been completely evident. M. Andral admits that, in such cases, the liquid filling the cavity is rapidly absorbed, but Dr. Stokes, in the true spirit of medical philosophy, makes this inquiry; "may not the phenomenon be explained, by supposing that the softened tuberculous matter had passed into, and filled the smaller bronchial tubes, from whence, owing to the great debility of the patient, it could not be expectorated?" Vast excavations, entirely empty, have been found in the lungs of people who had ceased to expectorate immediately before death.

In most patients, the phthisical sputa have a faint and nauseous smell; and, in them, the disease may go through its different stages and death supervene, without the sputal odour becoming more disagreeable: at other times, although long inodorous, it will, a few days before death, acquire an insupportable fœtor which is also perceived in the matter of the cavities. What, it has been asked, is the cause of this change of odour? That, because the fœtor of pus, which often arises from its contact with air, may occasionally depend on other circumstances, as its own qualities and the time it has remained in the cavity, the same should be true of the liquid contained in tuberculous excavations of the lung, is not a satisfactory explanation. From whatever cause it may proceed, however, it is a fatal symptom when occurring in a person whose expectoration was previously inodorous: it is far less unfavourable, at the disease's commencement.

Persons, whose sputa are insipid, it has been said, sink less rapidly into a state of emaciation; but experience does not altogether confirm this position. Many phthisical patients, who complained of the insupportable taste of their expectoration, nevertheless, sunk but slowly; others, on the contrary, died rapidly, though, in them, it was nearly insipid: few, in fine, have the mild and saccharine, or saline taste, described by authors, as symptomatic of pulmonary consumption: nor is it decided whether the true phthisical expectoration can ever be so acrid as to erode the parts with which it may come in contact.

M. Andral has seen several instances where phthisis went through all its stages, where, even after death, large excavations were found in the lung; and in which, notwithstanding, the expectoration furnished no diagnostic sign whatever. In support of this singular fact, he adduces four pathological histories. as we cannot, however willing, make room for these, we shall transcribe Dr. Stokes' remarks on them. Of the preceding cases, he says, the third only appears to me to prove the extraordinary proposition in question. In the others, the excavations were found empty; and the expectoration, in the two first, had the tubercular character in some degree: these cases were also, he adds, too short a time under observation, to warrant the drawing of any general conclusion.

Large excavations are sometimes found filled with a grey or reddish liquid which was not observed in the expectoration, although by this the existence of phthisis was clearly indicated. When pneumonia supervenes on the phthisical state, the sputa change their character altogether, and only show acute inflammation of the lung, except in a few cases where the two expectorations are blended. Many patients have a characteristic expectoration during the night and morning only; and, for the rest of the day, they do not expectorate, or, if they do, the sputa are purely catarrhal: others expectorate only at the close of the hectic exacerbations, while, during the paroxysm, their cough remains dry: attention to these circumstances may prevent error. Tuberculous excavations are thus analogous to many external ulcers, whose surfaces become dry, during the accession of an intermittent, and again moist at the end of the paroxysm. In a phthisical person, who had caries of one of the ribs, the expectoration diminished whenever the suppuration increased, and *vice versâ:* sometimes, also, it happens that, at intervals, the sputa cease to be characteristic, without any cause being assignable for this change: such intermittence of the expectoration frequently alternates with diarrhœa; when the dejections become very frequent, the sputa are either less abundant or altogether disappear.

Thus we have, in four distinct articles on the books which treat of the subject, endeavoured to instruct our readers in the principles and practice of stethoscopy which, from its manifest usefulness, we rejoice to find so successfully cultivated by many of our ablest physicians. We would encourge every one, therefore, who has not yet availed himself of this almost infallible means of detecting the seat, nature, and extent of thoracic disease, to instantly shake off his indifference, lay aside his prejudices, and begin using the stethoscope with attention and judgment; and, in so doing, we shall venture to assure him of advantages which the most sanguine expectation could scarcely have anticipated. Dr. Stokes' volume, which we have shown to be composed with great discrimination and accuracy, will furnish every beginner with a cheap, convenient, and exceedingly faithful introduction to the art of stethoscopy, while those of Professor Laënnec, Dr. Forbes, and M. Andral will prove excellent guides to him through the highest and most important inquiries to which the maladies of the chest and its organs may give rise. We only ask, in fine, that the stethoscope be tried by the laws of its own application; and, on this being done, we fearlessly assert, that it will be found to accomplish the desired ends with as much perfection and certainty as any other invention which the genius of wisdom has ever elaborated for the good of mankind.

Dr. Bennet's Dissertation, the title of which stands at the head of this article, has already been approved by its judges, as the ultimate test of the author's acquirements in the sciences which entitle a worthy aspirant to be crowned "summis in medicinâ honoribus:" as, however, it is not intended, at least in its present form, for the use of general readers, we propose confining ourselves, on this occasion, to a mere outline of his plan and to express our sentiments in regard to its execution.

We begin, then, with allowing the Doctor to state his object in his own words. "In dissertatione sequente," he says, p. 2, "ea phænomena tantum in unum collegi, quæ mihi ex usu in diagnosi affectionum pulmonalium instituenda videbantur, et iis cum affectionibus diversis quas indicant, arctius conjunctis, compendium principiorum doctrinæ tradere conatus sum, dum, quod ad descriptionem statuum morbosorum in his variis morbis pleniorem, lectorem ad opus Professoris Laënnec celebre ipsum rejeci." He proceeds next, under the head "DE RESPIRATIONE," to consider the manifestations of this function as they appear in health and disease: connected with the former, he describes the *respiratory murmur* and *puerile breathing*, and, with the latter, the *tracheal* or *bronchial*, and *cavernous* respiration, and the *crepitant*, *mucous*, *sonorous*, and *sibilant* wheezes. Under the head, DE VOCE, he estimates the importance of *pectoriloquy*, *bronchophony*, and *egophony*, as available pathological signs: and, under the last head, DE CORDE, "de insignibus indiciis corde explorando præbitis, non in animum venit curiose disserere; pauca enim, ex his sunt quibus tuto confidendum censet." From our examination of Dr. B's. Essay, we have been enabled to discover that he is a zealous and philosophic auscultator; but, at the same time, we can also perceive his having exercised a most prudent caution in restraining his enthusiasm where its indulgence might not have been appreciated: novelty, we all know, is always startling to those who have ceased to inquire; besides, "bis pueri senes," is a maxim which cannot be impugned.

We hail Dr. Bennet's inaugural Dissertation, in the construction of which, both his genius and his judgment must necessarily have been considerably fettered, as the harbinger of higher aims in the career of honourable renown; and it rejoices us to find that, since his admission to the Doctorate, he has published two works, by which, although translations, a fair estimate of his learning and talents may be formed: one of these is Bayle's Manual of Anatomy, the other, Professor Tiedemann's celebrated description of the Growth of the Fœtal Brain, both of which we shall ultimately analyze.

